# Robust control for a tracked mobile robot based on a finite-time convergence zeroing neural network

**DOI:** 10.3389/fnbot.2023.1242063

**Published:** 2023-09-20

**Authors:** Yuxuan Cao, Boyun Liu, Jinyun Pu

**Affiliations:** College of Power Engineering, Naval University of Engineering, Wuhan, China

**Keywords:** tracked mobile robot, trajectory tracking, finite-time convergence, zeroing neural network, robust

## Abstract

**Introduction:**

Since tracked mobile robot is a typical non-linear system, it has been a challenge to achieve the trajectory tracking of tracked mobile robots. A zeroing neural network is employed to control a tracked mobile robot to track the desired trajectory.

**Methods:**

A new fractional exponential activation function is designed in this study, and the implicit derivative dynamic model of the tracked mobile robot is presented, termed finite-time convergence zeroing neural network. The proposed model is analyzed based on the Lyapunov stability theory, and the upper bound of the convergence time is given. In addition, the robustness of the finite-time convergence zeroing neural network model is investigated under different error disturbances.

**Results and discussion:**

Numerical experiments of tracking an eight-shaped trajectory are conducted successfully, validating the proposed model for the trajectory tracking problem of tracked mobile robots. Comparative results validate the effectiveness and superiority of the proposed model for the kinematical resolution of tracked mobile robots even in a disturbance environment.

## 1. Introduction

At present, robots are being widely used in marine exploration (Fang et al., 2022; Wang et al., 2022), industrial manufacturing (Šegota et al., 2021; Truong et al., 2021), military applications (Bistron and Piotrowski, 2021; Rawat et al., 2021), and other fields. Tracked mobile robots (TMRs) show their wide adaptability and traffic ability to complex terrain (Gu et al., 2021). The demand for their motion autonomy and intelligence is increasing. Therefore, the control issue of trajectory tracking has been a research hotspot.

However, a TMR is a typical nonlinear system, and its model parameters change with its motion. In addition, the model is vulnerable to various interferences. The superposition of many factors poses a great challenge to the control algorithm. Therefore, a feasible solution with outstanding convergence performance as well as robustness to handle the nonlinear time-varying control issue of the TMR is imperative in practice. Numerous methodologies and techniques for addressing the tracking control issues of robot systems have been extensively studied and reported, including backstepping control (Ji et al., 2002; Gao et al., 2022; Sabiha et al., 2022), sliding mode control (Ahmed et al., 2021; Yin et al., 2021), fuzzy control (Lara-Molina and Dumur, 2021; Li et al., 2022), and neural network (Ding et al., 2018; Jin and Qiu, 2022).

Among various kinds of solutions, neural network approaches have shown huge advantages in terms of parallelism and easy implementation by hardware (Chen and Zhang, 2018). As a powerful approach for solving time-varying problems, the conventional zeroing neural network (CZNN) proposed in Zhang et al. (2002) has been thoroughly investigated in recent years (Miao et al., 2015; Xiao et al., 2017; Gerontitis et al., 2022; Sun et al., 2022; Zhang and Zheng, 2022). Ma et al. (2021) proposed a new ZNN model to solve the bound-constrained time-varying nonlinear equation, which has been applied to the mobile robot manipulator. Chen et al. proposed a multi-constrained ZNN. The application on the mobile manipulator for nonlinear optimization control demonstrated its physical effectiveness (Chen et al., 2021). Although CZNN can converge to the analytical solution with time, the convergence time is infinite in theory, which is impossible in reality. For an actual situation, the convergence time should be as short as possible. Moreover, CZNN is sensitive to noise and other disturbances. However, the system is susceptible to external disturbances and possible internal disturbances.

Many efforts have been made to address the shortcomings of CZNN. Hu et al. (2020) developed a noise tolerance ZNN model, which successfully tracked the desired path of the mobile manipulator with high accuracy under perturbation. Chen and Zhang (2018) proposed a robust ZNN model for solving the inverse kinematics problem of mobile robot manipulators . Luo et al. proposed a new hyperbolic tangent varying-parameter ZNN. Furthermore, trajectory tracking tasks of the mobile robot substantiate the outstanding convergence of hyperbolic tangent variant-parameter robust ZNN (HTVPR-ZNN) schemes (Luo et al., 2022). Chen et al. (2020) proposed a ZNN model with a super twisting algorithm that realized finite-time convergence and anti-disturbance, proving its effectiveness and superiority in the tracking control of the mobile robot manipulator. Lin et al. utilized a new design formula of noise resistance and finite-time convergence to establish a new ZNN. Compared with CZNN, the presented model was nonsensitive to various types of external disturbances (Xiao et al., 2019). Yan et al. (2019) proposed several improved ZNN models that allow nonconvex activation functions and have accelerated finite-time convergence.

However, the models and approaches reviewed above might potentially not be time-efficient and simultaneously robust for direct applications to a tracking control problem of TMR due to the requirement of timeliness as well as the influence of the disturbance environment. Moreover, it is worth pointing out that the robustness and finite convergence of ZNN models are related to the design of appropriate activation functions. The sign-bi-power function mentioned above endows ZNN with finite-time convergence, but it also contains a sign function, which may lead to singularity and discontinuity. Additionally, the performance under disturbance has not been not fully studied. Therefore, it is necessary to design a new activation function to obtain anti-interference and outstanding convergence.

Under the framework of the ZNN, a finite-time convergence ZNN, termed FCZNN, is proposed in this study. First, a new fractional evolution formula is designed to accelerate the convergence speed and enhance its robustness, which can converge to the desired trajectory within a finite-time under four common disturbances. To better demonstrate the contribution of this study, some existing models are introduced for comparison to highlight the main differences, and the corresponding comparison results are presented in Section 4.

The rest of this paper is organized into four sections. Section 2 presents a novel tracking control method based on FCZNN models for TMR. Section 3 validates the finite convergence and other properties. Section 4 illustrates the corresponding simulation results of the proposed method and presents some existing models for comparison. Section 5 concludes the entire paper.

Before ending this section, the main contributions of this study are summarized as follows:

- A new fractional exponential activation function is proposed in this study and investigated to solve the trajectory tracking issue. Compared with the tunable activation function, the singularity and sign function can be effectively avoided by reasonably selecting the design parameters.
- The finite-time convergence and robustness of the proposed FCZNN are validated theoretically based on the Lyapunov stability theory.
- Simulation experiments are conducted to present the verification and superiority of the FCZNN when compared with some existing models. Additionally, the validity of the theoretical analysis is confirmed based on the corresponding results.

## 2. Preliminaries

Since the actual situation is complicated, it is difficult to reflect it fully. Appropriate simplification is necessary. First, the main application scenario of our TMR is in a structured environment, such as indoors or on roads, and it can be analyzed on a two-dimensional plane. Furthermore, the difference in grounding pressure and the mass distribution of the TMR affect the kinematic model of the TMR. To simplify the kinematics model, some assumptions are declared for the TMR:

** Assumption 1**. *The TMR moves on the flat terrain with even tracking grounding pressure*.

** Assumption 2**. *The centroid of the TMR is located at the center of the robot*.

**Figure 1:**
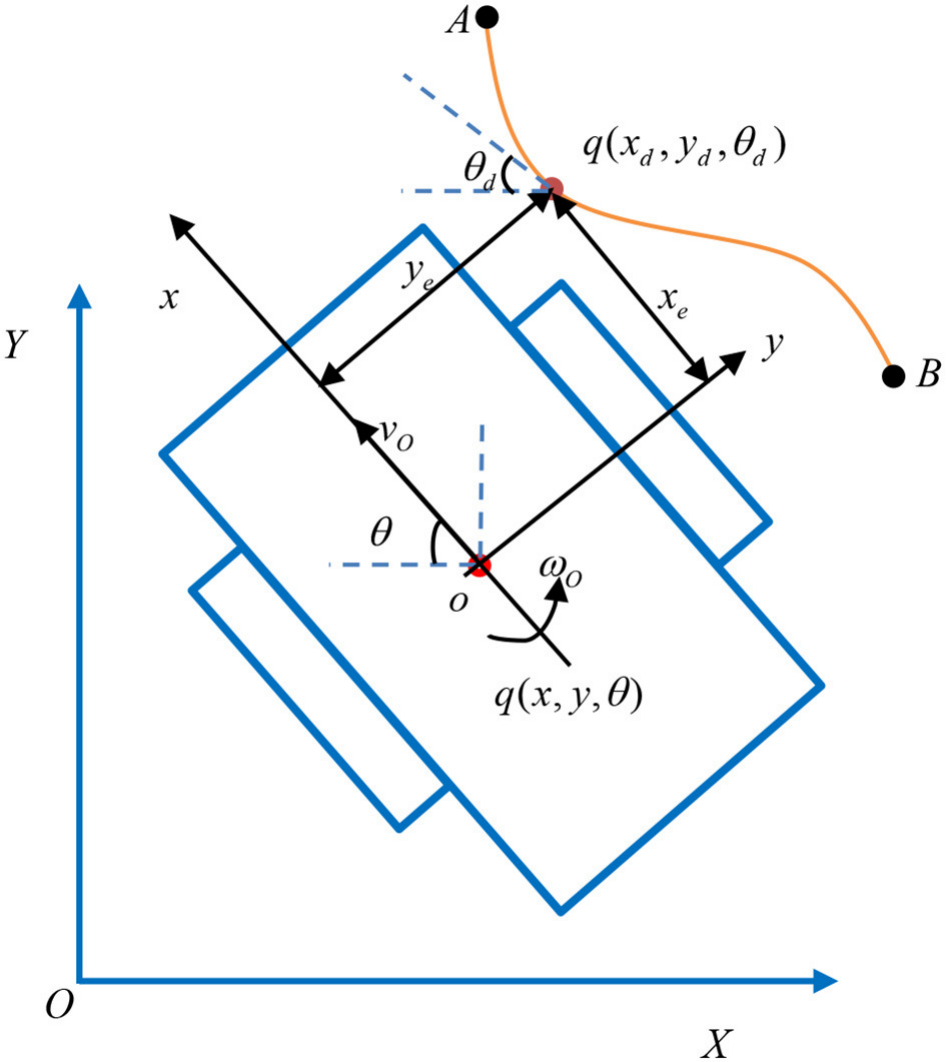
Schematic diagram of the motion of the TMR.

In the global *XOY* coordinate system, the schematic diagram of the motion of the TMR is presented in Figure 1. Some notations mentioned in Figure 1 are listed in Table 1.

**Table 1:** Notations in Figure 1.

**Notation**	**Meaning**
*xoy*	The coordinate system attached to the TMR
*q*(*x, y*, θ)	The actual position
*q*(*x*_*d*_, *y*_*d*_, θ_*d*_)	The desired position
*o*	The centroid of the TMR
θ	The heading angle of the TMR
*v*_*o*_	The velocity of the TMR
ω_*o*_	The angular velocity of the TMR

First, we introduce a model-free tracking control method for the TMR relying only on user-defined input and sensory output without knowing any information about the model parameters of the TMR. The kinematics model of the TMR is depicted as


(1) $$\begin{array}{l} \dot{q}\left(t\right)=J\left(\theta \right)u\left(t\right) \end{array}$$

where *q*(*t*) = [*x, y*, θ]^*T*^ is the generalized coordinates of the TMR, $\dot{q}\left(t\right)$ is the time-derivative of *q*(*t*) , *u*(*t*) = [*v*, ω]^*T*^ is the control input vector, and *J*(*θ*) = [cos *θ*, 0; sin *θ*, 0; 0, 1] is the full-rank velocity transformation matrix. To obtain the solution of the matrix equation, the FCZNN model is presented to solve this kind of a robot trajectory control issue.

A time-varying desired path equation *q*_*d*_(*t*) is offered for tracking using the TMR,


(2) $$\begin{array}{l} \dot{q_{d}}\left(t\right)=J\left(\theta_{d}\right)u_{d}\left(t\right) \end{array}$$

where ${\dot{q}}_{d}\left(t\right)$ denotes the time derivate of *q*_*d*_(*t*), *J*(*θ*_*d*_) = [cos *θ*_*d*_, 0; sin *θ*_*d*_, 0; 0, 1] is the desired full-rank velocity transformation matrix, and $u_{d}\left(t\right)={\left[v_{d},\omega_{d}\right]}^{T}$ is the desired control input vector. The mapping relation in real time *t* is expressed as *q*(*t*) → *q*_*d*_(*t*) . The mapping at the velocity level is shown as $\dot{q}\left(t\right)\to {\dot{q}}_{d}\left(t\right)$

The following error function is defined in the global coordinate system:


(3) $$\begin{array}{l} q_{e}\left(t\right)=q\left(t\right)-q_{d}\left(t\right) \end{array}$$

The error is generally defined in the coordinate system of then TMR; then, one has


(4) $$\begin{array}{l} e\left(t\right)=Tq_{e}\left(t\right) \end{array}$$

where $e\left(t\right)={\left[e_{x},e_{y},e_{\theta }\right]}^{T}$ and *T* = [cos, sin *θ*, 0; sin *θ*, − cos *θ*, 0; 0, 0, 1] is the coordinate transformation matrix, which converts the tracking error defined under the inertial coordinate system to the body coordinate system.

In view of the design rules of the ZNN, the following formula is given:


(5) $$\begin{array}{l} \frac{de\left(t\right)}{dt}=-\text{ΓΦ}\left(e\left(t\right)\right) \end{array}$$

where Φ(*e*(*t*)) denotes an activation function vector with various type, linear type, power type, etc. Theoretically, any monotonically increasing odd function can be the activation function candidate. Γ is a positive-definite matrix for scaling the convergence rate of the solve process. Based on the related derivate theory, Γ should be set as large as possible within the tolerance limit of the hardware. For ease of discussion, Γ is set as a diagonal matrix with the same element, that is, Γ = γ*I*, where *I* is the identity matrix. Additionally, Γ is a constant scalar-valued parameter matrix. Then,


(6) $$\begin{array}{l} ė\left(t\right)=-\gamma \text{Φ}\left(e\left(t\right)\right) \end{array}$$

where *γ* is the parameter that adjusts the convergence rate.

Moreover from (13), one promtly has


(7) $$\begin{array}{l} ė\left(t\right)=Ṫq_{e}\left(t\right)+T\dot{q_{e}}\left(t\right) \end{array}$$

## 3. Model design and theoretical analysis

In this section, a finite-time and robust unified framework synthesized by adopting a new activation function is proposed. The relative theorems and proofs about the corresponding features, namely, of finite-time convergence, global stability, and robustness in the disturbance environment, are explored to demonstrate the effectiveness of the proposed FCZNN model.

Considering (1), (6), and (7), one can obtain


(8) $$\begin{array}{l} T\left(J\left(\theta \right)u\left(t\right)-{\dot{q}}_{d}\left(t\right)\right)+Ṫ\left(q\left(t\right)-q_{d}\left(t\right)\right)=-\gamma \text{Φ}\left(e\left(t\right)\right) \end{array}$$

Evidently, the neural dynamics Equation (8) makes full use of the pose information and its derivate of the TMR, which contributes to solving the trajectory tracking control problem.

To demonstrate the anti-interference performance of the proposed FCZNN, some theorems about robustness are investigated in this section. Generally, the synthesized error caused by the disturbances is inevitable for any electronic system and neural dynamics. The synthesized error caused by hardware implementation off-set errors can be treated as dynamic non-disappearing noise in linear or sine form. The one caused by the instantaneous decline of power sources or other external disturbances can be regarded as dynamic disappearing noise in exponential form. Then, the implicit dynamic Equation (8) with the synthesized error is reformulated


(9) $$\begin{array}{l} T\left(J\left(\theta \right)u\left(t\right)-{\dot{q}}_{d}\left(t\right)\right)+Ṫ\left(q\left(t\right)-q_{d}\left(t\right)\right)=-\gamma \text{Φ}\left(e\left(t\right)\right)+W\left(t\right) \end{array}$$

where *W*(*t*) ∈ *R*^3^ denotes the synthesized error (could be constant or time-varying) with each entry *w*_*i*_(*t*) ≤ *w* for *i* = 1, 2, 3, where *w* ≥ 0 is an unknown constant.

### 3.1. Design of the FCZNN

As mentioned before, the choice of error evolution formula has a crucial influence on the characteristics of the system. Inspired by Xiao et al. (2017), a new fractional exponential activation function is proposed for constructing the error evolution formula.


(10) $$\begin{array}{l} \text{Φ}\left(x\right)=\kappa_{1}f^{p/p_{1}}\left(x,t\right)+\kappa_{2}f\left(x,t\right)+\kappa_{3}f^{p_{1}/p}\left(x,t\right) \end{array}$$

where *f*(*x, t*) is the set of increasing odd functions and design parameters *p* and *p*_1_ denote positive odd integer with *p* > *p*_1_, κ_1_ > 0, κ_2_ > 0 , κ_3_ > 0. Evidently, three terms of the activation function are odd functions the sum of the three terms is still a monotonically increasing odd function. For analysis, we define *f*(*x, t*) = *x*. Then, the error evolution formula is given as


(11) $$\begin{array}{l} \frac{de\left(t\right)}{dt}=-\gamma \left(\kappa_{1}e^{p/p_{1}}\left(t\right)+\kappa_{2}e\left(t\right)+\kappa_{3}e^{p_{1}/p}\left(t\right)\right) \end{array}$$

where *γ* is defined as before. The Equation (9) can be reformulated as


(12) $$\begin{array}{l} u\left(t\right)=J^{†}\left(\theta \right)T^{-1}\left[-\gamma \text{Φ}\left(e\left(t\right)\right)-Ṫ\left(q\left(t\right)-q_{d}\left(t\right)\right)+T{\dot{q}}_{d}\left(t\right)+W\left(t\right)\right] \end{array}$$

where *J*^†^(*t*) denotes the pseudo inverse of *J*(*t*).

**Figure 2:**
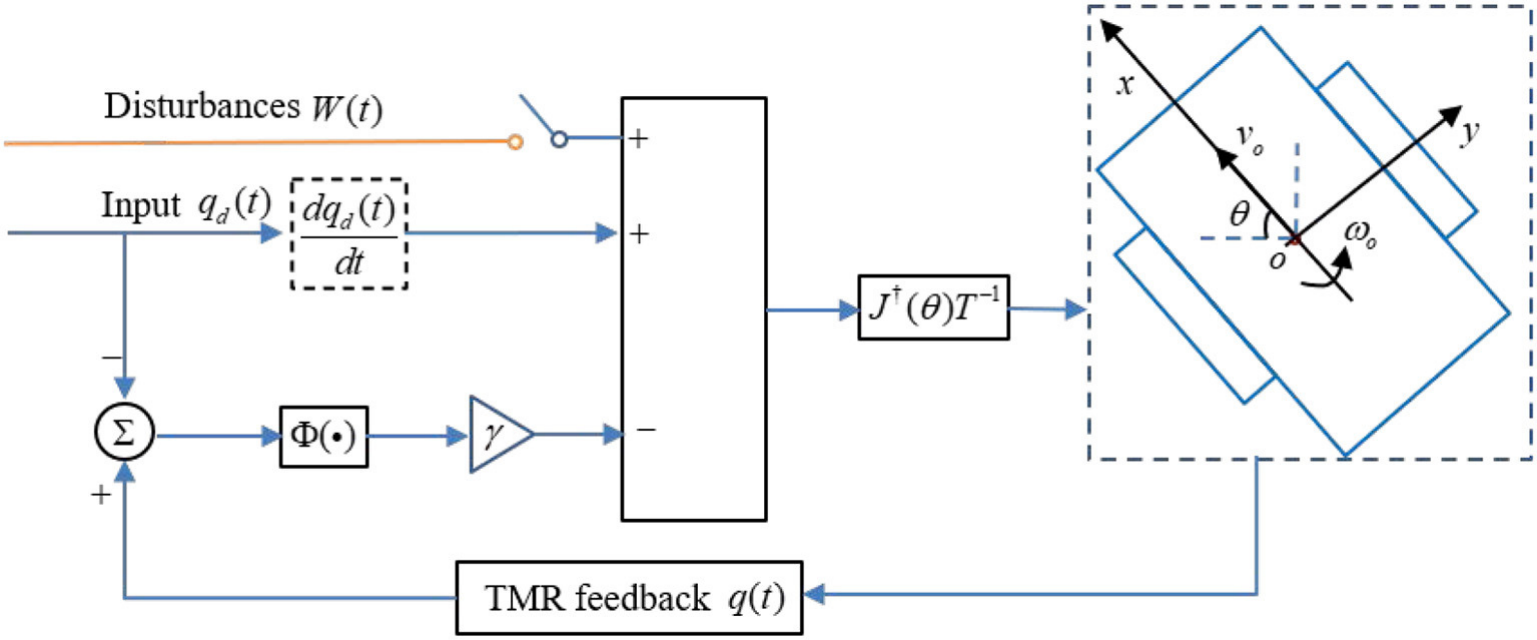
Block diagram of the FCZNN model with the possible disturbances for *W*(*t*) handling tracking control issue of the TMR.

The detailed algorithm description about the FCZNN model for the TMR tracking control issue is presented in Algorithm 1. The block diagram presented in Figure 2 demonstrates the principle of the control strategy.

**Algorithm 1:** Tracking control of the TMR via the FCZNN.

**Initialize:** TMR initial state vector combined velocity vector$\dot{q}\left(0\right)$; **Choose:** The tracking duration *T*_*f*_ and design parameters *γ* and κ_*i* = 1, 2, 3_ ; **Input:** The desired position *q*_*d*_(*t*) of tracking task;
1: **if** *t* < *T*_*f*_ **then**
2: **Calculate**: The desired path as ${\dot{q}}_{d}\left(t\right)$ ;
3: **Read**: The real time TMR actual position *q*(*t*);
4: **Calculate**: The control-signal by using neuron dynamic equation
5:
$u\left(t\right)=J^{†}\left(\theta \right)T^{-1}\left[-\gamma \text{Φ}\left(e\left(t\right)\right)-Ṫ\left(q\left(t\right)-q_{d}\left(t\right)\right)+T{\dot{q}}_{d}\left(t\right)+W\left(t\right)\right]$
6: **Update**: The TMR position in the next moment
7: **Output**: The actual trajectory *q*(*t*)
8: **else**
9: **Stop**: TMR trajectory tracking task finished.
10: **endif**

To illustrate the details of the proposed model, the *i*th (*i* = 1, 2, 3) neuron of the FCZNN is given below.


(13) $$\begin{array}{l} {\dot{q}}_{i}=-\gamma \phi \left(e_{i}\right)+w_{i}-\sum_{j=1}^{3}\left({Ṫ}_{ij}e_{j}+T_{ij}{\dot{q}}_{dj}\right) \end{array}$$

where ${\dot{q}}_{i}$, ${\dot{q}}_{dj}$ denote the *i*th element of $\dot{q}$, ${\dot{q}}_{d}$, respectively, and Ṫ_*ij*_, *T*_*ij*_ are the (*i, j*)th element of Ṫ and *T*.

**Figure 3:**
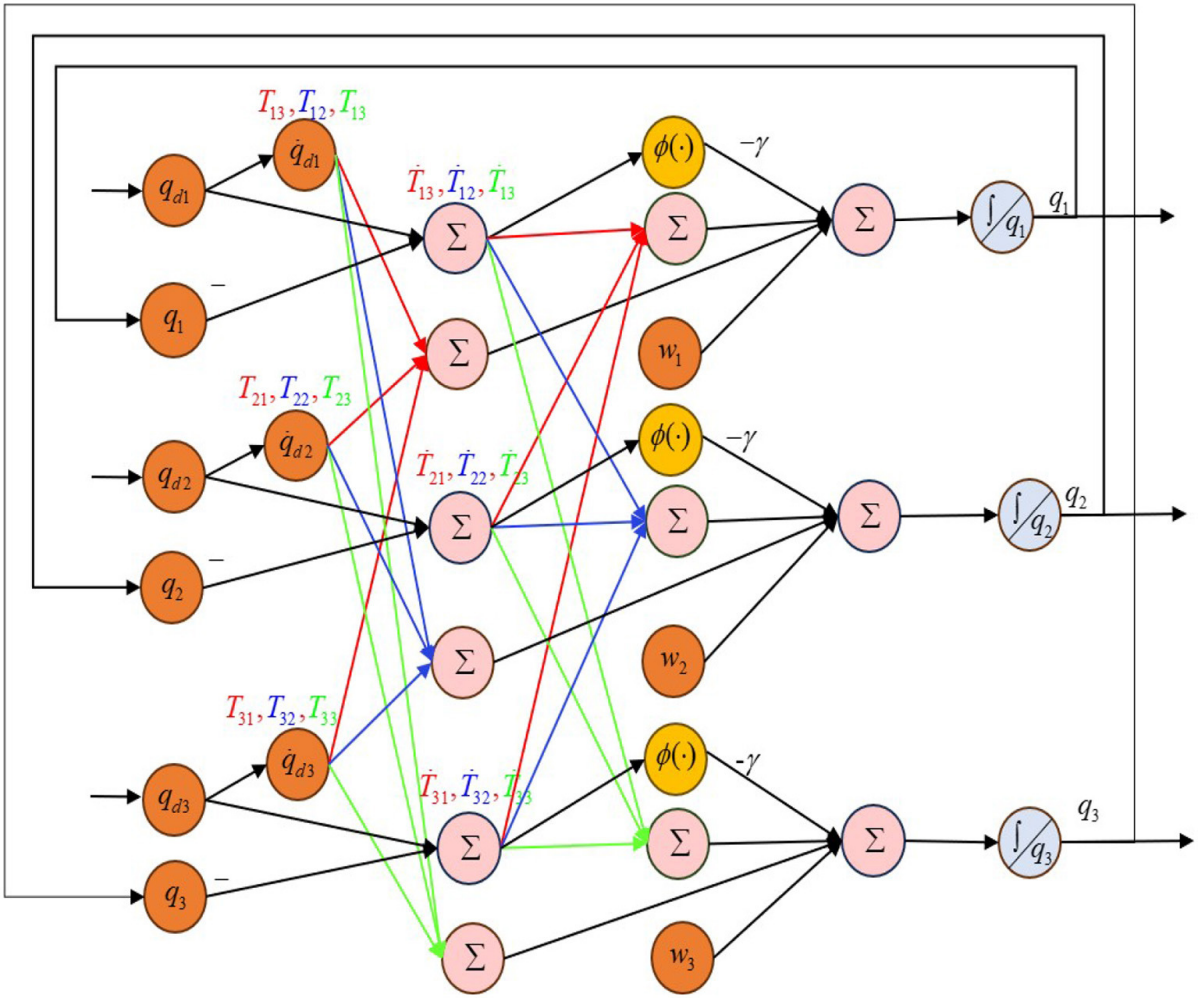
Neural topology of the proposed FCZNN model.

Based on (13), the neural topology structure of the proposed FCZNN model is presented in Figure 3.

### 3.2. Convergence analysis

#### 3.2.1. Global stability analysis

**Theorem 1**. *If a monotonically increasing odd function Φ(·) is taken as the activation function, the output will globally converge to the desired trajectory *q*_*d*_(*t*) of the model (9) with a random generated initial state *q*(0)*.

*Proof:* To prove the global convergence of the model (9), the following Lyapunov function candidate is presented as


(14) $$\begin{array}{l} L\left(t\right)=\frac{||e\left(t\right)|{|}_{2}^{2}}{2}=\frac{e^{T}\left(t\right)e\left(t\right)}{2} \end{array}$$

where ||·||_2_ denotes the two norm of a vector. Considering (6), the derivate of the above function is


(15) $$\begin{array}{l} \begin{array}{c} \dot{L}\left(t\right)=e^{T}\left(t\right)\frac{e\left(t\right)}{dt}\\ \;=-\gamma e^{T}\left(t\right)\text{Φ}\left(e\left(t\right)\right)\\ \;=-\gamma \overset{m}{\underset{i=1}{\sum }}e_{i}\phi \left(e_{i}\left(t\right)\right) \end{array} \end{array}$$

where *e*_*i*_(*t*) is the *i*th element of *e*(*t*), *ϕ*(*e*_*i*_(*t*)) is the *i*th element of Φ(*e*(*t*)), and *m* = 3 represents the number of model subsystems. Since the activation function is an odd function, the following relationship exists:


(16) $$\begin{array}{l} e_{i}(t)\phi (e_{i}(t))=\{\begin{array}{l} >0,\;if\;e_{i}(t)\neq 0\\ =0,\;if\;e_{i}(t)=0 \end{array}. \end{array}$$

According to the Lyapunov stability theory, the system is asymptotically stable at moment *t* with $\dot{L}\left(t\right)<0$ guaranteed. Considering (16), we have


(17) $$\begin{array}{l} \dot{L}(t)=-\gamma \sum_{i=1}^{m}e_{i}\phi (e_{i}(t))=\{\begin{array}{c} =0\;if\;e_{i}(t)=0\\ <0,\;if\;e_{i}(t)\neq 0 \end{array},\;t\in [0,\;+\infty ) \end{array}$$

                                                ⎕

Equation (17) demonstrates that $\dot{L}\left(t\right)$ is negative finite. Based on the Lyapunov stability theory, the system will gradually stabilize with time, the error equation will converge to 0, and the corresponding input will converge to the analytical solution. The proof of global convergence is thus completed.

Theorem 1 indicates that the system residual error converges to 0, which means that the TMR can track the desired position with time. The evolution formula proposed in this study demonstrates that the tracking task of a desired path can converge in the finite time. Next, the finite-time convergence of the FCZNN is proved below.

#### 3.2.2. Finite-time convergence analysis

** Theorem 2**. *Considering the novel activation function (10) for the error function e(t), e(t) can converge to 0 in finite time T_f_. T_f_ satisfies the following inequality:*


(18) $$\begin{array}{l} T_{f}\leq \{\begin{array}{l} \frac{1}{2\gamma \kappa_{2}\left(\frac{\left(p+p_{1}\right)}{2p_{1}}-1\right)}\ln\left(\frac{\kappa_{1}+\kappa_{2}}{\kappa_{2}L{\left(0\right)}^{1-\frac{p+p_{1}}{2p_{1}}}+\kappa_{1}}\right)+\frac{\ln\left(1+\frac{\kappa_{2}}{\kappa_{3}}L{\left(0\right)}^{1-\frac{\left(p+p_{1}\right)}{2p}}\right)}{2\gamma \kappa_{2}\left(\frac{\left(p+p_{1}\right)}{2p}-1\right)},L(t)\geq 1\\ \frac{\ln\left(1+\frac{\kappa_{2}}{\kappa_{3}}L{\left(0\right)}^{1-\frac{\left(p+p_{1}\right)}{2p}}\right)}{2\gamma \kappa_{2}\left(\frac{\left(p+p_{1}\right)}{2p}-1\right)},L(t)<1 \end{array} \end{array}$$

*Proof:* Firstly, the maximum initial value element of the error function is depicted as $e^{+}(0)=\max_{i=1,2,3}\{|e_{i}(0)|\}$. The following relationship holds true: $-|e^{+}\left(t\right)|\leq |e_{i}\left(t\right)|\leq |e^{+}\left(t\right)|$ for *t* ≥ 0 and *i* = 1, 2, 3 , which reveals that *e*_*i*_(*t*) converges to 0 when *e*^+^(*t*) is equivalent to 0. Moreover, ė^+^(*t*) = - *γ* Φ (*e*^+^(*t*)).


(19) $$\begin{array}{l} \begin{array}{c} \dot{L}\left(t\right)=2{ė}^{+}\left(t\right)e^{+}\left(t\right)\\ \;=-2\gamma \text{Φ}\left(e^{+}\left(t\right)\right)e^{+}\left(t\right)\\ \;=-2\gamma \left(\kappa_{1}L{\left(t\right)}^{\left(p+p_{1}\right)/2p_{1}}+\kappa_{2}L\left(t\right)+\kappa_{3}L{\left(t\right)}^{\left(p_{1}+p\right)/2p}\right) \end{array} \end{array}$$

For simplicity, we define 2*γκ*_1_ = β_1_ , 2*γκ*_2_ = β_2_ , 2*γκ*_3_ = β_3_ , *a* = (*p* + *p*_1_)/2*p*_1_ , and *b* = (*p* + *p*_1_)/2*p*. In view of the precondition, *a* > 1 , 0 < *b* < 1 . Then, $\dot{L}\left(t\right)=-\left(\beta_{1}L^{a}\left(t\right)+\beta_{2}L\left(t\right)+\beta_{3}L^{b}\left(t\right)\right)$.

Inequality (18) is proved below. The following two situations exist:

CASE I: When *L*(*t*) ≥ 1,


(20) $$\begin{array}{l} \dot{L}\left(t\right)\leq -\beta_{1}L^{a}\left(t\right)-\beta_{2}L\left(t\right) \end{array}$$

Inequality (20) can be transformed as


(21) $$\begin{array}{l} \frac{dL\left(t\right)}{\beta_{3}L^{a}\left(t\right)+\beta_{2}L\left(t\right)}\leq -dt \end{array}$$

Integrating both sides of (21) from 0 to t, we can obtain


(22) $$\begin{array}{l} \;L(t)=\;\\ \{\begin{array}{l} \leq \exp(-\beta_{2}t){\left(L^{1-a}(0)+\frac{\beta_{1}}{\beta_{2}}-\frac{\beta_{1}}{\beta_{2}}\exp((1-a)\beta_{2}t)\right)}^{\frac{1}{1-a}},if\;0\leq t<t_{1}\\ =1,\;if\;t=t_{1} \end{array} \end{array}$$

where *t*_1_ denotes the convergence time to 0 for $L^{1-a}(0)\text{=}\max_{i=1,2,3}\{e_{{}_{i}}^{1-a}(0)\}.$

Let *L*(*t*) = 1 ,


(23) $$\begin{array}{l} t_{1}=\frac{1}{\left(a-1\right)\beta_{2}}\ln\;\frac{\beta_{1}+\beta_{2}}{\beta_{2}L^{1-a}\left(0\right)+\beta_{1}}. \end{array}$$

CASE II: When *L*(*t*) ≤ 1,


(24) $$\begin{array}{l} \dot{L}\left(t\right)\leq -\left(\beta_{2}L\left(t\right)+\beta_{3}L^{b}\left(t\right)\right). \end{array}$$

Inequality (24) can be converted to


(25) $$\begin{array}{l} \frac{dL_{2}\left(t\right)}{\beta_{2}L_{2}\left(t\right)+\beta_{3}L_{2}^{b}\left(t\right)}\leq -dt. \end{array}$$

Integrating the above differential inequality from 0 to t, we have


(26) $$\begin{array}{l} \;L(t)=\;\\ \{\begin{array}{l} \leq \exp(-\beta_{2}t){\left(L^{1-b}(0)+\frac{\beta_{3}}{\beta_{2}}-\frac{\beta_{3}}{\beta_{2}}\exp((1-b)\beta_{2}t)\right)}^{\frac{1}{1-b}},if\;0\leq t<t_{2}\\ =0,\;if\;t=t_{2} \end{array}. \end{array}$$

Similarly, *t*_2_ satisfies the following equality:


(27) $$\begin{array}{l} t_{2}=\frac{\ln\;\left(1+\frac{\beta_{2}}{\beta_{3}}L^{1-b}\left(0\right)\right)}{\beta_{2}\left(1-b\right)}. \end{array}$$

where *t*_2_ denotes the convergence time to 0 for *L*(*t*) ≤ 1 , and $L^{b}(0)\text{=}\max_{i=1,2,3}\{e_{i}^{b}(0)\}.$

In summary, the upper bound of convergence time *T*_*f*_ satisfies


(28) $$\begin{array}{l} T_{f}\leq \{\begin{array}{l} \frac{1}{(a-1)\beta_{2}}\ln\left(\frac{\beta_{1}+\beta_{2}}{\beta_{2}L^{1-a}(0)+\beta_{1}}\right)+\frac{\ln\left(1+\frac{\beta_{2}}{\beta_{3}}L^{1-b}(0)\right)}{\beta_{2}(1-b)},L(t)\geq 1\\ \frac{\ln\left(1+\frac{\beta_{2}}{\beta_{3}}L^{1-b}(0)\right)}{\beta_{2}(1-b)},\;L(t)<1 \end{array}. \end{array}$$

Note that (28) can be rewritten in the form of (18). The proof is thus completed.    □

### 3.3. Robustness analysis

The CZNN has been proven to converge to the desired result in the disturbance-free case. However, in the practical situation, the disturbance cannot be avoided. The tracking error may arise in the presence of the disturbance. In this section, the steady-state error is given base on the Lyapunov theory.

** Theorem 3**. *Consider tracking control issue (1) of the TMR. Suppose that an FCZNN model is polluted by the additive bounded error w_i_(t) with w_i_(t) ≤ w (constant or time-varying disturbance), where w is positive constant, starting from the arbitrary initial position q(0), the steady-state tracking error of the FCZNN model (9) yields the following equality*:


(29) $$\begin{array}{l} \underset{t\to +\infty }{\lim}{\left\|e(t)\right\|}_{2}<\sqrt{m}{\left(\frac{w}{\gamma \kappa_{3}}\right)}^{p_{1}\;/p}. \end{array}$$

*where all the parameters in the inequality have been defined before*.

*Proof:* Provided that the additive disturbances exist in the FCZNN model, its *i*th dynamical subsystem corresponding to the error function in the FCZNN model is given by


(30) $$\begin{array}{l} {ė}_{i}\left(t\right)=-\gamma \phi \left(e_{i}\left(t\right)\right)+w_{i} \end{array}$$

Similar to Theorem 1, a Lyapunov function is defined first to address the global convergence of the proposed FCZNN model.


(31) $$\begin{array}{l} L\left(t\right)=\frac{p}{p+p_{1}}e_{i}{\left(t\right)}^{\frac{p_{1}+p}{p}} \end{array}$$

Obviously, *L*(*t*) is an even function, *L*(*t*) ≥ 0 . Taking derivation for *L*(*t*) , we have


(32) $$\begin{array}{l} \dot{L}(t)=e_{i}{\left(t\right)}^{p_{1}/p}{ė}_{i}(t)\\ \text{ =}\left[-\gamma \left(\kappa_{1}e_{i}{\left(t\right)}^{p/p_{1}}+\kappa_{2}e_{i}(t)+\kappa_{3}e_{i}{\left(t\right)}^{p_{1}/p}\right)+w_{i}\right]e_{i}{\left(t\right)}^{p_{1}/p}\\ \text{ =}-\gamma \;\kappa_{3}{\left(e_{i}{\left(t\right)}^{p_{1}/p}-\frac{w_{i}}{2\gamma \kappa_{3}}\right)}^{2}+\frac{w_{i}{}^{2}}{4\gamma \kappa_{3}}-\\ \left(\gamma \kappa_{1}e_{i}{\left(t\right)}^{(p^{2}+p_{1}{}^{2})/pp_{1}}+\gamma \kappa_{2}e_{i}{\left(t\right)}^{(p+p_{1})/p}\right) \end{array}$$

Suppose that $e_{i}\left(t\right)\geq {\left(w/\gamma \kappa_{3}\right)}^{p/q}$ , the first two terms hold $-\gamma \kappa_{3}{\left(e_{i}{\left(t\right)}^{q/p}-\frac{w_{i}}{2\gamma \kappa_{3}}\right)}^{2}+\frac{{w_{i}}^{2}}{4\gamma \kappa_{3}}<0$ .

Based on this, we obtain the following analysis about Equation (32). There are two situations.

1) If solution error $e_{i}\left(t\right)\geq {\left(w/\gamma \kappa_{3}\right)}^{p/q}$ holds true, one can readily obtain that $\dot{L}\left(t\right)<0$ . In the sense of the Lyapunov theory, the system becomes stable gradually with time.

2) If solution error $e_{i}\left(t\right)<{\left(w/\gamma \kappa_{3}\right)}^{p/p_{1}}$ holds true, the sign of $\dot{L}\left(t\right)$ might be positive or negative. Even in the worst-case scenario, we consider $\dot{L}\left(t\right)>0$ , which indicates that *e*_*i*_(*t*) will increase; ${\left(w_{i}/\gamma \kappa_{3}\right)}^{p/p_{1}}$ does not exceed the upper bound ${\left(w/\gamma \kappa_{3}\right)}^{p/p_{1}}$ for $\dot{L}\left(t\right)<0$ when $e_{i}\left(t\right)\geq {\left(w_{i}/\gamma \kappa_{3}\right)}^{p/p_{1}}$ .

Recalling that $||e\left(t\right)|{|}_{2}=\sqrt{\sum_{i=1}^{m}e_{i}^{2}\left(t\right)}$, one can readily draw the conclusion that $\underset{t\to +\infty }{\lim}||e(t)|{|}_{2}<\sqrt{m}{\left(\frac{w}{\gamma \kappa_{3}}\right)}^{p_{1}/p}.$ The proof is thus completed.                                    ⎕

It is worth pointing out that Theorem 2 presents that the steady-state solution error can be arbitrarily small by increasing or reducing the fractional value.

** Theorem 4**. *In the case of $e_{i}\left(t\right)\geq {\left(w_{i}/\gamma \kappa_{3}\right)}^{p/p_{1}}$ , starting from any initial value *q*(0) , the actual trajectory *q*(*t*) tracks the desired position *q*_*d*_(*t*) in finite time *T*_*f*_ for the FCZNN model (9) with constant noises. *T*_*f*_ satisfies the following equality:*


(33) $$\begin{array}{l} T_{f}\leq \frac{1}{\left(a-1\right)\beta_{2}}\ln\;\frac{\beta_{1}+\beta_{2}}{\beta_{2}L^{1-a}\left(0\right)+\beta_{1}} \end{array}$$

*where the parameter in (33) is predefined in Theorem 2*.

*Proof:* A Lyapunov function *L*(*t*) = (*e*^+^(*t*))^2^ is defined; the derivate of *L*(*t*) is demonstrated


(34) $$\begin{array}{l} \begin{array}{l} \dot{L}(t)=2{\dot{e}}^{+}(t)e^{+}(t)\\ \;=2\left(-\gamma \Phi \left(e^{+}(t)\right)+w_{i}\right)e^{+}(t)\\ \;=(-2\gamma \kappa_{1}L{\left(t\right)}^{(p+p_{1})/2p_{1}}-2\gamma \kappa_{2}L(t)-2\gamma \kappa_{3}L{\left(t\right)}^{(p_{1}+p)\;2p}+\\ 2w_{i}e^{+}(t)) \end{array} \end{array}$$

Then, $\dot{L}\left(t\right)$ is rewritten as $\dot{L}\left(t\right)=-\left(\beta_{1}L^{a}\left(t\right)+\beta_{2}L\left(t\right)+\beta_{3}L^{b}\left(t\right)\right)+2w_{i}e^{+}\left(t\right)$ Considering Theorem 3, if $e_{i}\left(t\right)\geq {\left(w/\gamma \kappa_{3}\right)}^{p/p_{1}}$ holds true (i.e., $w_{i}\leq \beta_{3}e^{+}{\left(t\right)}^{p_{1}/p}/2$), one can have


(35) $$\begin{array}{l} \begin{array}{c} 2w_{i}e^{+}\left(t\right)\leq \beta_{3}e^{+}{\left(t\right)}^{\left(p+p_{1}\right)/p}\\ \;=\beta_{3}L{\left(t\right)}^{\left(p+p_{1}\right)/2p} \end{array} \end{array}$$

Then, (34) is reformulated as


(36) $$\begin{array}{l} \dot{L}(t)=-(\beta_{1}L^{a}(t)+\beta_{2}L(t)+\beta_{3}L^{b}(t)+we^{+}(t))\\ \;\leq (-\beta_{1}L^{a}(t)-\beta_{2}L(t)-\beta_{3}L^{b}(t)+\beta_{3}L^{b}(t))\\ \text{ =}-\beta_{1}L^{a}(t)-\beta_{2}L(t) \end{array}$$

Based on the discussion in Theorem 2, *T*_*f*_ satisfies (33). Then, the proof is completed.

## 4. Numerical experiments

The numerical experiments are conducted in this section to demonstrate the finite-time convergence and robustness of the FCZNN model with disturbance considered. The CZNN is adopted for comparison.

During the initialization of the algorithm, the initial position vector is set to be *q*(0) = *q*_*d*_(0) + Δ*o* . The vector Δ*o* is the off-set between an actual position and the desired position in the Cartesian space. Δ*o* = (0, 1, 0) is set in the simulation. The predefined design parameter is set to be *γ* = 10, and we keep κ_*i*_ = 10 for *i* = 1, 2, 3. Moreover, *p* and *p*_1_ are set to be 9 and 3 separately. In the application, the TMR is applied to track an eight-shaped path. The reference trajectory for TMR is given by


(37) $$\begin{array}{l} \{\begin{array}{c} x_{d}=h_{1}\sin(h_{2}t),\\ y_{d}=h_{1}\sin(h_{3}t), \end{array}t\in [0,T] \end{array}$$

where [*h*_1_, *h*_2_, *h*_3_] = [10, 0.01, 0.05]. Then, we have

**Remark 1**. *The scope of eight-shaped reference trajectory can be adjusted by changing the value of h*_1_, *that is*, (*x*_*d*_, *y*_*d*_) ⊂ {(*x*_*d*_, *y*_*d*_)|−*h*_1_ ≤ *x*_*d*_ ≤ *h*_1_, −*h*_1_ ≤ *y*_*d*_ ≤ *h*_1_}.


(38) $$\begin{array}{l} \{\begin{array}{l} {\dot{x}}_{d}=h_{1}h_{2}\cos(h_{2}t),\;{\dot{y}}_{d}=h_{1}h_{3}\cos(h_{3}t),\\ {\ddot{x}}_{d}=-h_{1}h_{2}^{2}\sin(h_{3}t),\;{\ddot{y}}_{d}=-h_{1}h_{3}^{2}\sin(h_{3}t),\\ v_{d}=\sqrt{{\dot{x}}_{d}^{2}+{\dot{y}}_{d}^{2},}\\ \theta_{d}=\arctan\;2({\dot{x}}_{d},{\dot{y}}_{d}). \end{array} \end{array}$$

The path-tracking task duration is set to be 150s as the initialization. Meanwhile, the general tracking error is expressed as the two norm of the error vector.


(39) $$\begin{array}{l} ||E|{|}_{2}=\sqrt{{\left(x-x_{d}\right)}^{2}+{\left(y-y_{d}\right)}^{2}+{\left(\theta -\theta_{d}\right)}^{2}}. \end{array}$$

### 4.1. Finite-time convergence validation without disturbance

**Figure 4:**
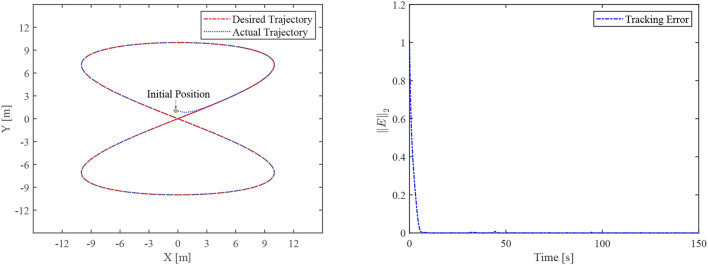
The tracking performance of the FCZNN without disturbance.

The simulative results of the FCZNN without disturbance are shown in Figure 4. Figure 4 presents the tracking performance for the TMR to track the eight-shaped path, which shows that the actual trajectory moves toward the desired trajectory and demonstrates the tracking error during the tracking task, validating the finite-time convergence with global stability. The tracking error decreases directly from the maximum value, which indicates that the error is related to the setting of the initial position because the error of the robot in the initial position is the maximum, consistent with the theoretical analysis.

### 4.2. Robustness verification

In general, disturbances are unavoidable for any electronic system and neural dynamics, mainly including internal and external disturbances. Internal disturbances are caused by hardware implementation off-set errors, which can be viewed as dynamic disturbances in linear or sinusoidal form. External disturbances are caused by instantaneous changes in power or external shock among other reasons, which can be regarded as the disturbance that disappears exponentially.

The disturbances considered in this study are shown in Table 2, including four different common disturbances.

**Table 2:** The disturbances forms.

**No**.	**Disturbance forms**	**Expression**
1	Constant form	*w*_*i*_ = 1
2	Line form	*w*_*i*_ = 0.01*t
3	Sine form	*w*_*i*_ = *sint*
4	Exponential decay form	*w*_*i*_ = exp(−*t*)

**Figure 5:**
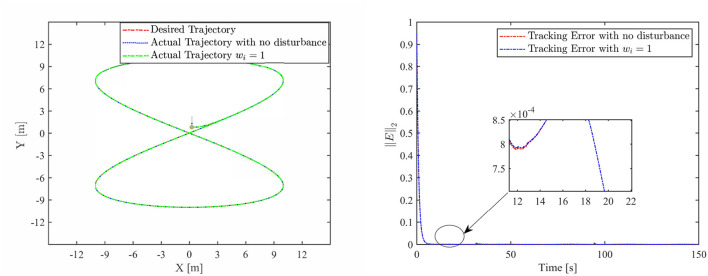
The tracking performance of the FCZNN model with disturbance *w*_*i*_ = 1 .

**Figure 6:**
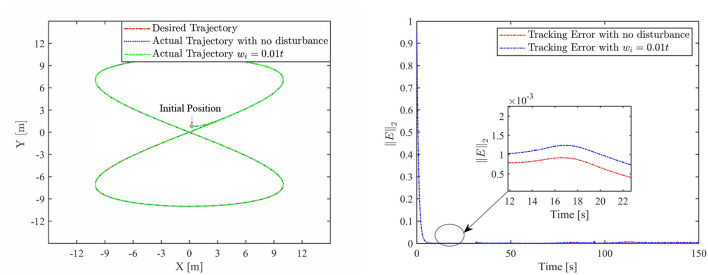
The tracking performance of FCZNN with disturbance *w*_*i*_ = 0.01 ∗ *t* .

**Figure 7:**
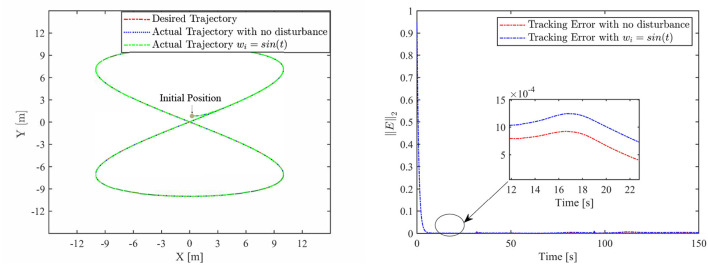
The tracking performance of the FCZNN model with disturbance *w*_*i*_ = *sin*(*t*) .

**Figure 8:**
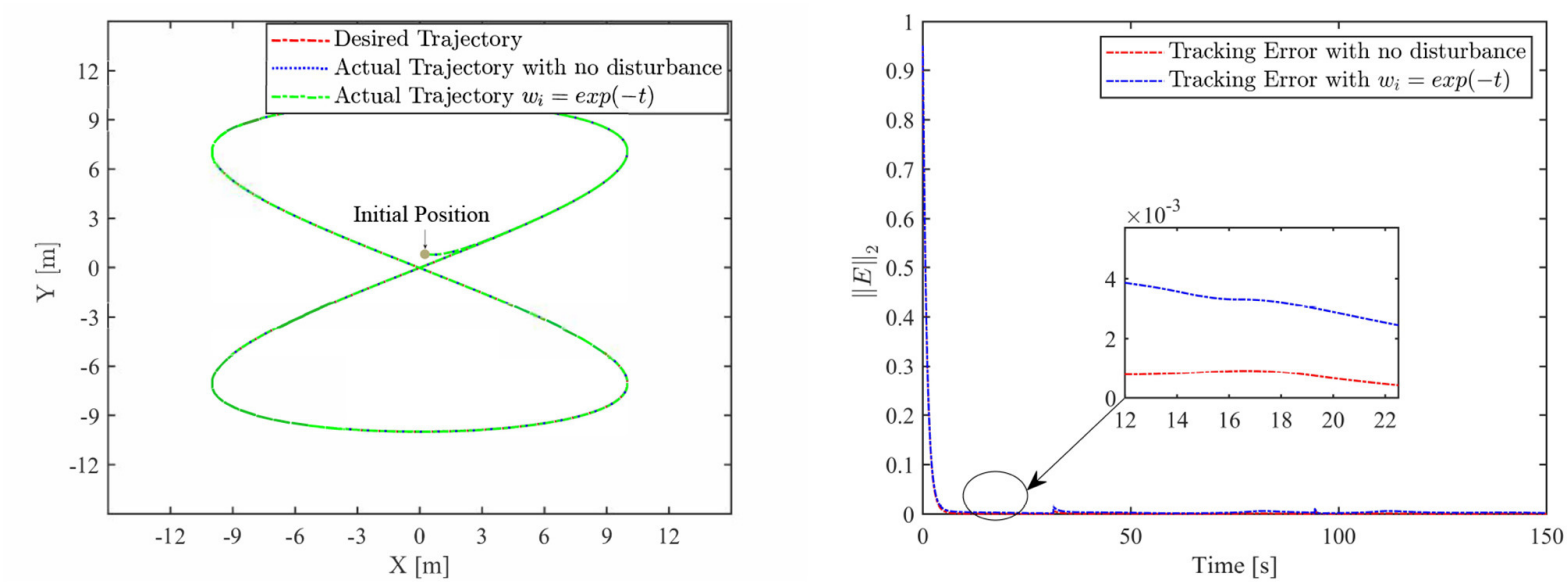
The tracking performance of the FCZNN model with disturbance *w*_*i*_ = exp(−*t*) .

Th motion results of the TMR tracking an eight-shaped path synthesized by the FCZNN model are shown in Figures 5–8. Figure 5 shows that under constant value perturbations, the FCZNN model still has an excellent effect on the tracking error of the trajectory, indicating that it has a better suppression effect on constant value perturbations. Figures 6, 7 present that under linear or sine-form perturbations, there is still room for improvement in the suppression of the FCZNN model. Figure 7 illustrates that perturbations in the exponential decay form have a larger impact on the system at the moment they occur, unlike linear and sinusoidal perturbations.

Combing in the above figures, in the disturbance environment, the FCZNN model can still guarantee finite-time convergence. That is, in a disturbance environment, the TMR can still track the desired trajectory. Certainly, the convergence time is longer than that in Figure 4. The previous analysis illustrates that the tracking effect can be further enhanced by changing the parameters. In addition, we notice that the convergence time in the case of constant interference is longer than that in the case of time-varying disturbance. The upper limit of the time-varying disturbance is 1, and the time-varying disturbance is 0 at the beginning of the numerical experiment. Hence, the FCZNN model can track the desired trajectory faster.

### 4.3. Comparison with existing models

**Figure 9:**
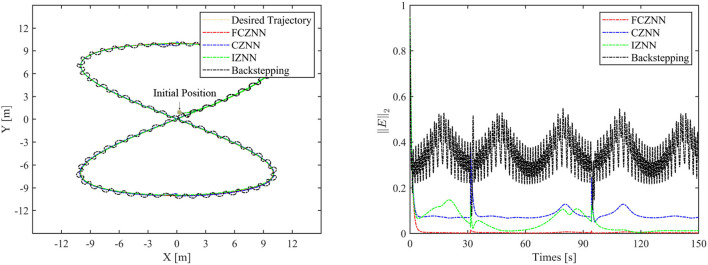
Simulated motion results with *w*_*i*_ = 1.

**Figure 10:**
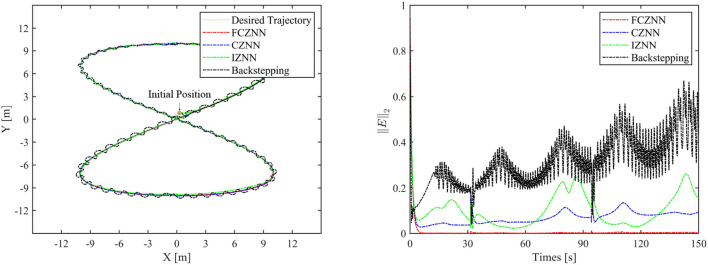
Simulated motion results with *w*_*i*_ = 0.01*t*.

**Figure 11:**
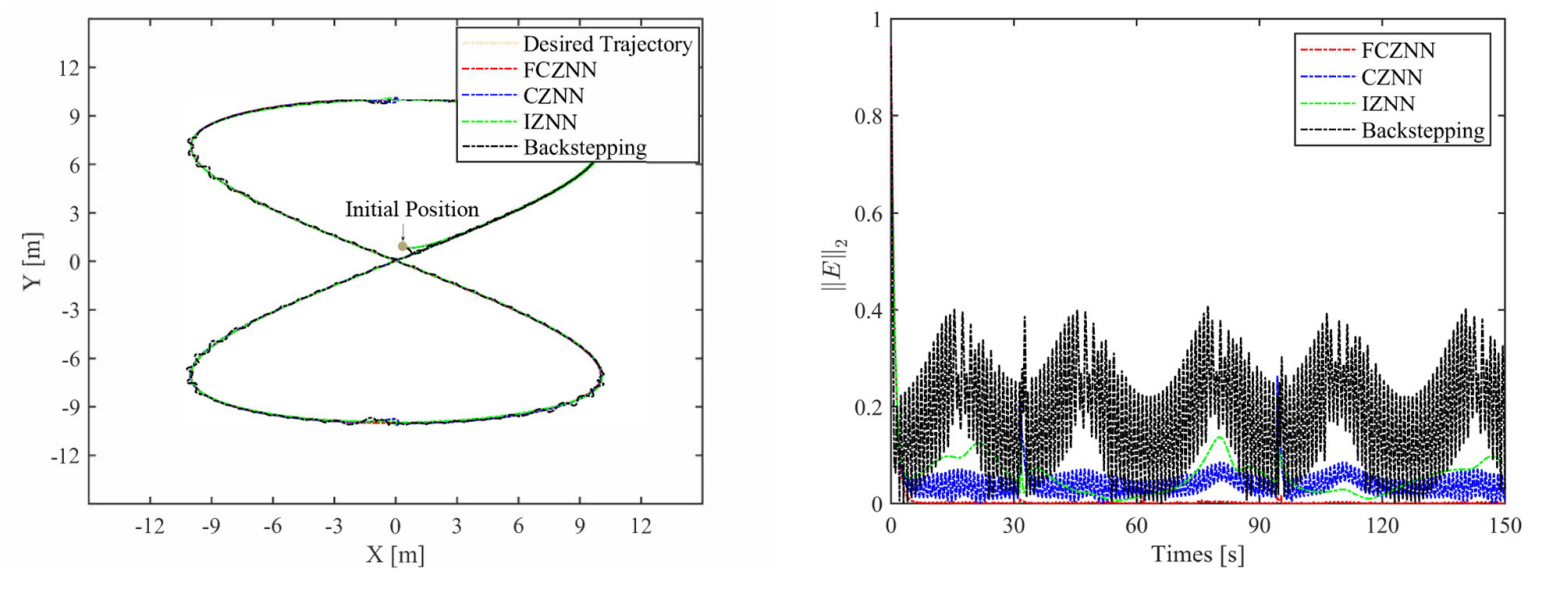
Simulated motion results with *w*_*i*_ = *sin*(*t*).

**Figure 12:**
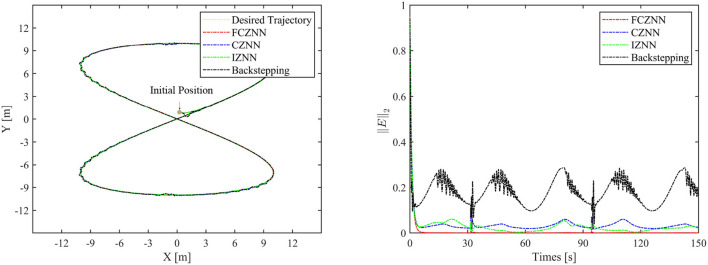
Simulated motion results with *w*_*i*_ = exp(−*t*).

To verify the efficacy and superiority of the FCZNN model, comprehensive comparisons with existing neural network models are presented in this section, including the CZNN (Miao et al., 2015; Xiao et al., 2017) and integration-enhanced ZNN (IZNN) (Chen and Zhang, 2018; Xiao et al., 2019). Moreover, the classical backstepping control Hao et al. (2017) is introduced for comparison as well. Figures 9–12 show the comparison results of various models with different disturbances. Clearly, all four methods are able to complete the task of trajectory tracking, but the quality differs considerably.

For solving the inverse kinematics problem of the mobile robot, the CZNN model with the disturbances can be depicted as the following dynamic equation:


(40) $$\begin{array}{l} A\left(t\right)u\left(t\right)-{\dot{q}}_{d}\left(t\right)=-\gamma e\left(t\right)+W\left(t\right) \end{array}$$

The convergence feature of the CZNN model without disturbance has been investigated broadly and is neglected in this study. Without loss of generality, parameters *γ* and κ_*i*_ for *i* = 1, 2, 3 are kept the same.

The blue line in Figures 9–12 demonstrates the tracking performance of the CZNN model and its tracking error, showing that this model is sensitive to disturbances, especially the three time-varying disturbances. Figure 9 shows that the maximum tracking error of this model is much higher than that of the FCZNN and IZNN models. Generally, the tracking error of the CZNN model does not converge to be 0 during the entire tracking duration. Therefore, the CZNN model is not suitable for application in the disturbance environment.

The IZNN model has been presented and investigated as an alternative for solving the inverse kinematics problem of mobile robot manipulators; this model with disturbances can be depicted as the following dynamic equation:


(41) $$\begin{array}{l} A\left(t\right)u\left(t\right)-{\dot{q}}_{d}\left(t\right)=-\gamma e\left(t\right)-\text{λ}\int \text{Φ}e\left(t\right)dt+W\left(t\right) \end{array}$$

Readily, the simulation results present that the performance of the IZNN model is enhanced compared to that of the CZNN model. Figures 9–11 present that the IZNN model is nonsensitive to constant and exponential decay disturbances, but it cannot deal with sine or linear disturbances effectively. It does not meet our requirements.

Backstepping control is the classical method for solving the inverse kinematics problem of the mobile robot. However, the simulation results present its failure in achieving satisfactory results in an interference environment. Specifically, its tracking trajectory is not smooth, not to mention its tracking error. Details about backstepping control will, therefore, not be discussed in the paper.

Figures 9–12 illustrate that the proposed FCZNN model exhibits anti-disturbance performance with four common forms of disturbances suppressed for solving the inverse kinematics problem of the TMR compared with the existing two models and backstepping control. In addition, comparisons with other models or methods with the corresponding results shown in Figure 9 substantiate the robust property and finite convergence of the proposed FCZNN model, which are absent in both the CZNN and IZNN models.

Based on the above simulation results and analysis, we can draw the conclusion that the proposed FCZNN model has excellent and inherent noise and disturbance canceling ability accompanied by finite-time convergence, which enables it to be more suitable for practical applications of the TMR with noises and disturbances.

## 5. Conclusion

An FCZNN model was proposed in this study as a solution to the TMR tracking control . Different from the CZNN model, a new activation function was incorporated with the FCZNN model. Some theorems of finite-time convergence and strong robustness were mathematically validated. Simulation experiments were conducted to verify the superiority and effectiveness of the proposed FCZNN model in comparison with the CZNN, IZNN, and backstepping control. Furthermore, the application to TMR kinetic control presented its practical significance.

Future work lies in extending the kinematic analysis by considering multiple physical constraints and developing a complete experimental environment equipped with the real TMR for practical application of the FCZNN model. The extension of the FCZNN model to other similar mechanisms is an interesting, open, and challenging future direction for this research. Moreover, developing ZNN models that consider obstacle avoidance and saturation constraints to enable the TMR with active obstacle avoidance or developing novel saturation-allowed activation functions to adapt to practical requirements is an interesting research direction.

## Data availability statement

The original contributions presented in the study are included in the article/supplementary material, further inquiries can be directed to the corresponding author.

## Author contributions

YC and BL: data curation and validation. YC, BL, and JP: conceptualization and formal analysis. YC: methodology, software, and writing—original draft. JP: supervision. All authors have read and agreed to the published version of the manuscript.
